# Myasthenia Gravis Triggered by a COVID-19 Infection: A Case Report and Literature Review

**DOI:** 10.7759/cureus.59538

**Published:** 2024-05-02

**Authors:** Alexandra Mincă, Dragos I Mincă, Amalia L Calinoiu, Valeriu Gheorghiță, Claudiu C Popescu, Adina Rusu, Alexandra M Cristea, Dana G Mincă

**Affiliations:** 1 Public Health, Carol Davila University of Medicine and Pharmacy, Bucharest, ROU; 2 Internal Medicine, Agrippa Ionescu Emergency Clinical Hospital, Bucharest, ROU; 3 Anatomy, Carol Davila University of Medicine and Pharmacy, Bucharest, ROU; 4 Rheumatology, “Dr. Ion Stoia” Center of Rheumatic Diseases, Bucharest, ROU; 5 Infectious Diseases, Agrippa Ionescu Emergency Clinical Hospital, Bucharest, ROU; 6 Infectious Diseases, Carol Davila University of Medicine and Pharmacy, Bucharest, ROU; 7 Internal Medicine and Rheumatology, Carol Davila University of Medicine and Pharmacy, Bucharest, ROU; 8 Pulmonology, Marius Nasta Institute of Pneumology, Bucharest, ROU; 9 Pulmonology, Carol Davila University of Medicine and Pharmacy, Bucharest, ROU

**Keywords:** sars-cov-2, coronavirus disease 2019, autoimmune diseases, covid-19, myasthenia gravis (mg)

## Abstract

Myasthenia gravis (MG) is an autoimmune disease that induces skeletal muscle weakness, affecting different muscle groups. Severe acute respiratory syndrome coronavirus 2 (SARS-CoV-2), the cause of coronavirus disease 2019 (COVID-19), became both a diagnostic and a therapeutic challenge during the pandemic. The effects of COVID-19 are not only limited to the acute symptoms but also to the post-infectious sequelae. We present the case of a 30-year-old Caucasian woman, with no significant medical history, who presented to the emergency room with acute respiratory failure. The patient tested positive for SARS-CoV-2 with a rapid antigen test and during hospitalization developed a myasthenic crisis, ultimately being diagnosed with seropositive MG.

## Introduction

Myasthenia gravis (MG) is an autoimmune disease that induces skeletal muscle weakness, affecting different muscle groups [1]. The antibodies against the components of the neuromuscular junction are the cause of this pathology, the most frequently involved being the acetylcholinesterase receptor (AChR). Antibodies against AChR (AChR-ab) are present in approximately 80% of MG patients [2]. In 4% of cases, patients develop antibodies against muscle-specific kinase (MuSK-ab), and in 2% of cases, they develop low-density lipoprotein receptor-related protein 4 (Lrp4-ab) [3-6]. The effect of these antibodies is a decrease in the number of AChRs and structural alterations of the neuromuscular endplate.

Representing the most common acquired pathology of neuromuscular transmission, the prevalence of MG is between 150 and 200 cases/1,000,000 inhabitants, with an annual incidence rate in Europe of 4.1 to 30 cases/1,000,000 persons [7]. Women are more frequently affected between the ages of 15 and 64 years, and after this age, the incidence is higher in men [6]. Because of the existing genetic predisposition, the risk of first-degree relatives of MG patients to develop the disease is 4.5% [8]. The symptoms of MG include weakness and fatigue in the neck, eyes, or limb muscles. In addition, patients may experience difficulties in breathing, speaking, and chewing [9].

Severe acute respiratory syndrome coronavirus 2 (SARS-CoV-2), the cause of coronavirus disease 2019 (COVID-19), became both a diagnostic and therapeutic challenge during the pandemic. The effects of this infection are still being studied as they are limited not only to the acute symptoms but also to the long-term consequences [10].

We present the case of a 30-year-old Caucasian woman, with no significant medical history, who presented to the emergency room with acute respiratory failure. The patient tested positive for SARS-CoV-2 with a rapid antigen test and during hospitalization developed a myasthenic crisis. She was ultimately diagnosed with seropositive MG.

## Case presentation

A 30-year-old Caucasian woman was admitted via the emergency room in the Department of Internal Medicine at the University Hospital of Bucharest for severe dyspnea, fever (38.7°C), and coughing in the last seven days, which progressively worsened. The patient had no significant family or personal medical history. Her obstetric past included one pregnancy 18 months before with cesarean delivery and without complications. She was not receiving any chronic medication. Moreover, she was not vaccinated against SARS-CoV-2 and had no personal history of this infection.

**Figure 1:**
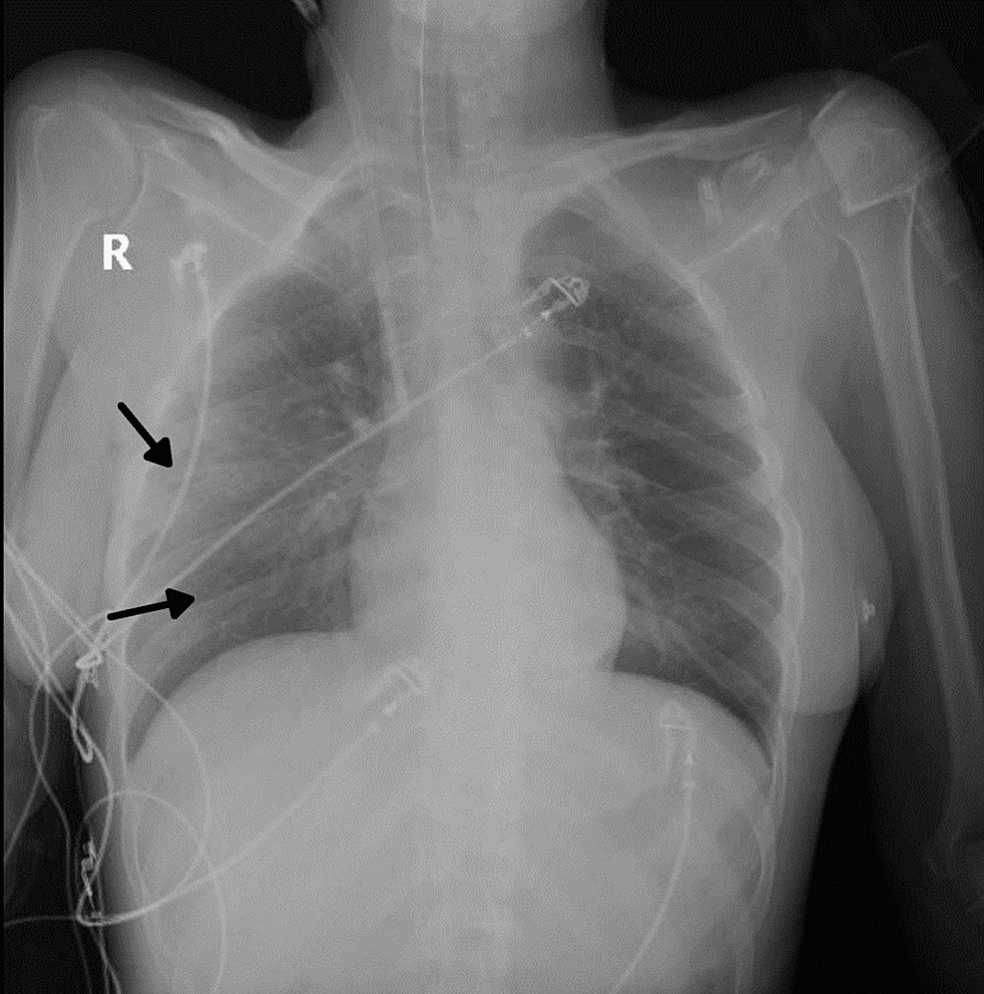
Chest X-ray posteroanterior view showing ground-glass opacities (arrows) in both the middle and lower lobes of the right lung.

At admission, the patient presented perioral and extremity cyanosis, tachypnea, polypnea, marked respiratory effort, asymmetric crepitant rales, blood oxygen saturation on pulse oximetry of 64% without oxygen supply and 90% with oxygen (10 L/minute), blood pressure of 120/65 mmHg, and heart rate of 100 beats/minute with a regular rhythm. In the emergency room, she tested positive for SARS-CoV-2 with a rapid antigen test. The chest X-ray indicated ground-glass opacities in both the middle and lower lobes of the right lung (Figure 1). Laboratory findings at admission revealed leukocytosis (15.8/μL; normal = <11.8/μL) with predominance of neutrophils, mild thrombocytopenia (123/μL; normal = >179/μL), high C-reactive protein (64.45 mg/L; normal = <5.0 mg/L), and high serum interleukin 6 (202.7 pg/mL; normal = <6.4 pg/mL).

The patient was admitted to the intensive care unit with severe acute respiratory failure due to SARS-CoV-2 bronchopneumonia. Initially, she was treated with high-flow nasal oxygenation therapy (peak inspiratory flow of 25 L/minute, fraction of inspired oxygen of 70%); intravenous remdesivir (200 mg in the first day, 100 mg for the next four days); intravenous ampicillin/sulbactam (1,000 mg/500 mg/day); intravenous pantoprazole (40 mg/day); oral n-acetylcysteine (600 mg/day), intravenous vitamin B1 (100 mg/day), vitamin B6 (250 mg/day), and vitamin C (750 mg/day); subcutaneous enoxaparin (4,000 IU/day); and intramuscular dexamethasone (8 mg/day). Her evolution was favorable both clinically and biologically, with improving symptoms, a decrease in the oxygen therapy requirement, remission of leukocytosis, and inflammatory biological syndrome during the first four days of hospitalization. Thus, she was transferred to the Department of Internal Medicine.

On day five, the patient presented an episode of psychomotor agitation alternating with drowsiness, associated with dysarthria and followed by dysphonia, dysphagia, and tetraparesis. To exclude an acute cerebral pathology, a brain magnetic resonance imaging study was performed without notable findings. The neurology consult noted the following: decreased muscle strength in all limbs, the inability to flex the head from a supine position, tetraparesis, dysphonia, dysarthria, frontalis orbicularis oculis 2/5 Medical Research Council (MRC), mild convergent strabismus, mild right eyelid ptosis with fluctuations of severity during the day, no sensitivity deficit, and normal osteotendinous reflexes. At this moment, the patient recalled that 18 months ago, after giving birth, she had noticed a slight decrease in muscle strength in the lower limbs and difficulties in lifting her head from the pillow.

**Figure 2:**
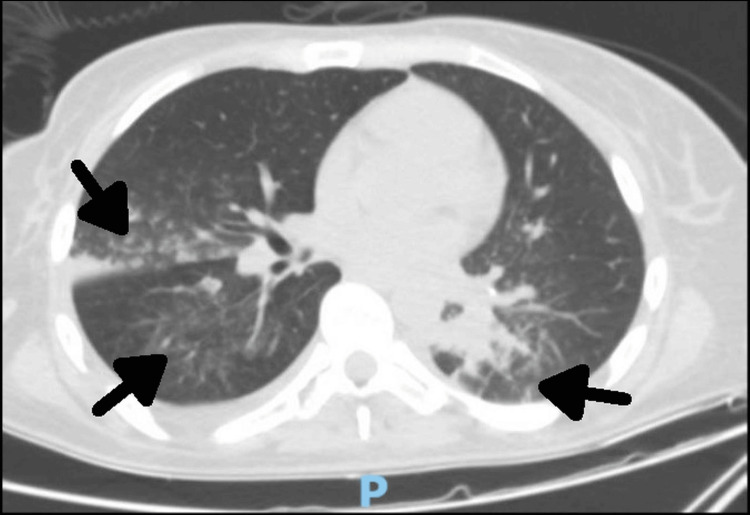
Computed tomography scan of the thorax showing ground-glass opacities (arrows) with superimposed interlobular septal thickening and intralobular septal thickening displaying the crazy paving appearance.

**Figure 3:**
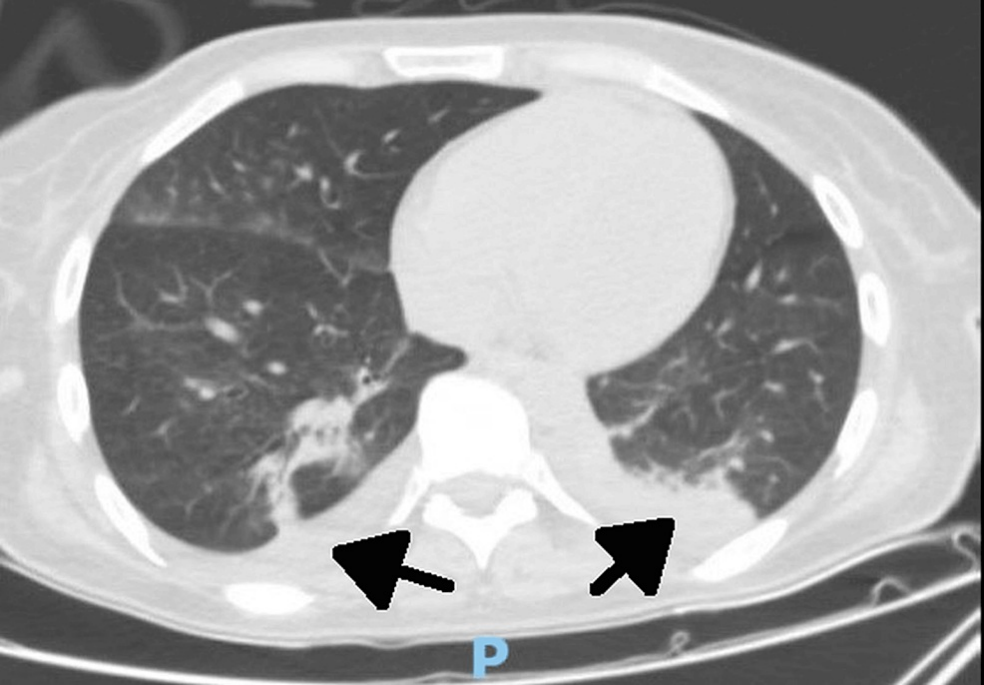
Computed tomography scan of the thorax showing bilateral pleural effusion (arrows).

During this time, even if in the first four days of hospitalization her evolution was favorable, as the patient’s condition worsened rapidly, she was readmitted to the intensive care unit where she developed acute hypercapnic respiratory failure and hypercapnic encephalopathy requiring intubation and mechanical ventilation. Biologically, the recurrence of leukocytosis with a predominance of neutrophils was observed. Computed tomography (CT) of the chest showed normal size and density of the thyroid (thymoma was excluded). At the lung level, areas of alveolar condensation and alveolitis were present in the dorsal segment of the right upper lobe, lateral to the middle lobe with the appearance of lamellar atelectasis in the posterior segment of the right lower lobe (Figure 2). In addition, the area of alveolar consolidation was also observed with apical and posterior air bronchogram in the left lower lobe, associating areas of alveolitis in the apical, lateral, and anteromedial segments due to the occupation by fluid secretions of the lobar bronchus and segmental branches associated with bilateral pleural effusion (15 mm on the right and 8 mm on the left; Figure 3).

Considering the strictly motor deficit symptomatology, generalized distribution, and fluctuation of severity during the day, the hypothesis of MG class IV B Osserman triggered by SARS-CoV-2 infection was raised. Thus, blood samples for both AChR-ab and MuSK-ab were collected. Plasmapheresis was immediately initiated and a total of five sessions were administered. The evolution was favorable. After two sessions of plasmapheresis, the patient was able to breathe without support and was extubated. After completion of plasmapheresis sessions and remission of the infectious episode, she received treatment with prednisone (1.5 mg/kg/day for three weeks, followed by a gradual decrease of doses by 5 mg every two weeks), intravenous pantoprazole (40 mg/day), oral vitamin D (2,000 U/day), oral calcium supplements (1,000 mg/day), and pyridostigmine (540 mg/day).

Meanwhile, the result of the blood samples collected during the patient’s second intensive care unit admission revealed an increased serum titer of AChR-ab (55.6 nmol/L, normal = <0.25 nmol/L). Therefore, based on clinical manifestations and autoimmunity tests, the patient was diagnosed with seropositive MG.

The evolution of symptoms under treatment was favorable with an improvement of muscle strength after one week of treatment (head elevation 4/5 MRC, frontalis orbicularis oculi 5/5 MRC, holding arms up for 50 seconds, holding lower limbs up for 40 seconds on right and 60 seconds on left). Additionally, after two weeks of treatment, immunosuppressive therapy was initiated with azathioprine (initial dose of 50 mg/day in the first week, increased to 75 mg/day in the second week). Subsequent assessments showed remission of symptoms after one month of treatment (head flexion 5/5 MRC, orbicularis oculi 5/5 MRC, holding arms raised more than 60 seconds, holding lower limbs raised more than 60 seconds).

## Discussion

In this case, the importance of the early diagnosis of MG was vital. Even if during the first days of the disease the evolution of the SARS-CoV-2 infectious episode was favorable, five days later, through the onset or exacerbation of MG, the patient’s condition deteriorated rapidly, a fact that endangered her life.

The diagnosis of autoimmune MG is based on the variety of symptoms, the presence and type of antibodies, the age of onset, and the thymic pathology correlation [11].

The clinical classification of MG consists of ocular myasthenia and generalized myasthenia, the second being divided, depending on the severity of the symptoms, into mild, moderate, and severe. Generalized myasthenia represents the involvement of any muscle group other than ocular muscles [12].

MG diagnosis is primarily clinical. Thus, a patient with MG manifestations should be tested for specific MG antibodies (AChR-ab, MuSK-ab), and if the result is positive, according to the literature, no further confirmation is necessary [13].

Unfortunately, laboratory results can be delayed; hence, in life-threatening situations, such as in the case described above, a faster diagnostic method is needed, which could be the therapeutic test. Our patient’s response to plasmapheresis and immunosuppressive and pyridostigmine treatment was favorable. She was able to breathe without support after the second plasmapheresis session. Moreover, at the one-year follow-up, she did not develop any myasthenic crisis and the muscle weakness improved.

Both viral and bacterial infections can be triggering factors for new-onset MG or myasthenic crises in an MG patient. Among the viruses involved in triggering MG are human immunodeficiency virus (HIV), poliomyelitis virus, or Epstein-Barr virus (EBV) [14,15].

Concerning COVID-19, the reported neurological manifestations included a wide spectrum, for example, anosmia, ageusia, consciousness disorder, dizziness, neuropathy, myopathy, Guillain-Barré syndrome, and neuromuscular disorders [16].

At the moment, few cases of new-onset MG triggered by both COVID-19 infection and the vaccine against this virus have been reported in the literature [17-26]. In 2020, Restivo et al. published an article in which three cases of AChR-ab-positive MG after SARS-CoV-2 infection were reported. In all three cases, as well as in the case of our patient, the specific symptoms of MG appeared five to seven from the onset of febrile episodes [21].

Regarding autoimmune diseases, the pregnancy period has different effects, with sexual hormones being incriminated as a trigger for worsening symptoms [27]. The postpartum period was identified as a risk factor for the onset of MG in a population-based cross-sectional study in Norway and the Netherlands [28]. Furthermore, considering the patient’s reports about the muscle weakness that appeared after giving birth, we can discuss the hypothesis that the SARS-CoV-2 infection could have been the triggering factor of a latent postpartum MG, but owing to the lack of clinical and paraclinical data from that period exists, we cannot affirm this.

## Conclusions

The presented case underlines the importance, sometimes vital, of the early diagnosis of MG. This article is one of the few reported cases of new-onset seropositive MG after SARS-COV-2 infection. We underline the need for validating these results in larger studies and for practitioners to be aware of and report such cases. Viral infections and, in particular, COVID-19 infection, through mechanisms still incompletely elucidated, can potentially be trigger factors for autoimmune diseases, as may have been the situation in the case presented above.
